# Relationship Between Training Frequency and Training Session Duration on Vitality in Recreational Runners: A Cross-Sectional Study

**DOI:** 10.3390/jfmk9040209

**Published:** 2024-10-27

**Authors:** Adrián Varela-Sanz, Marcos Mecías-Calvo, Erika Borrajo, Iker Muñoz-Pérez

**Affiliations:** 1 Performance and Health Group, Physical and Sports Education Department, Faculty of Sport Sciences and Physical Education, University of A Coruna, 15179 A Coruna, Spain; adrian.varela.sanz@udc.es; 2 Faculty of Teacher Training, University of Santiago de Compostela, 27001 Lugo, Spain; 3 Faculty of Psychology and Education, University of Deusto, 48007 Bilbao, Spain; borrajo.erika@deusto.es (E.B.); iker.munoz@deusto.es (I.M.-P.)

**Keywords:** running, life satisfaction, mental health, dose-response, training volume

## Abstract

**Background**: Running can improve health status from a biopsychosocial perspective. However, isolation strategies, like the COVID-19 pandemic-induced lockdown, produce deleterious effects on both health status and sport performance. The aim of our study was to investigate recreational runners’ sporting habits, subjective vitality (SV), and well-being after the COVID-19 pandemic-induced lockdown. **Methods**: After data filtration, 5542 recreational runners (74.5% men and 25.5% women, >18 years) were selected for further analyses. The participants answered preliminary questions regarding sporting habits and completed the validated Spanish version of the Subjective Vitality as a Dynamic Reflection of Well-Being questionnaire for assessing their SV after lockdown. **Results**: Subjective vitality scores did not differ between men and women, nor between age groups (*p* = 0.41 and *p* = 0.11, respectively). Subjective vitality was greater with weekly training frequency up to 5 days/week, where this enhancement plateaued, while average training session duration was positively related to SV, stabilizing at 91–120 min/session (*p* < 0.001 for both). **Conclusions**: There is a dose–response relationship between both weekly training frequency and training session duration, and mental health benefits in recreational runners. Further longitudinal studies are needed in order to determine the optimal dose–response relationship for simultaneously enhancing mental health outcomes and running performance in recreational runners, especially regarding weekly training frequency, training session duration, and exercise intensity.

## 1. Introduction

Recreational running is one of the most popular physical activities worldwide, as indicated by the increasing number of participants in running events (by around 58%) in the last decade [1]. Running can be considered a natural sporting activity inherent to the human species, and its effectiveness in maintaining or enhancing healthy fitness has been widely demonstrated [2]. Further, running also plays an important role in people’s health status at biological, psychological, and social levels, which is known as the biopsychosocial model [3,4]. This model considers health and disease as dynamic events based on the interactions of the three above-mentioned levels, which determines human functioning and, therefore, health status [5,6]. From this perspective, physical activity is considered as a complex, dynamic, and multidimensional process in which biological, psychological, and social aspects are interrelated and affected by individual differences [7,8].

The coronavirus disease (COVID-19) outbreak was declared a global pandemic by the World Health Organization (WHO) in March 2020, forcing governments to adopt prevention measures in order to slow the spread of COVID-19 (i.e., social distancing and limitation of social interactions, wearing of surgical masks, school and sport facilities closures, cancellation of sport events and, in most countries, an obligatory home quarantine) [9,10]. These measures differed between countries regarding the severity of the pandemic in each specific region. For instance, Spain and Italy, two of the most affected countries by the pandemic, adopted more restrictive strategies compared to France or Switzerland, which could affect the population’s health status, as well as their physical activity levels and sedentary behavior, in a different manner [11]. Concerning this, the unprecedented challenging situation generated by COVID-19 produced a negative impact on both social and psychological outcomes, which some have suggested have received less attention compared to the biological responses to the pandemic [12].

Most scientific evidence indicates that pandemic-related circumstances such as isolation strategies (e.g., home confinement) have negatively affected people’s health and well-being, especially when physical activity levels and sports practice are considered [13,14,15,16,17,18,19]. In this regard, recent reviews focusing on physical activity levels in the general population during the COVID-19 pandemic showed that moderate-to-vigorous physical activity decreased during lockdown, although overall physical activity levels were not substantially modified [20]. Furthermore, it was also suggested that simultaneously increasing sedentary time and decreasing physical activity led to psychological distress during the pandemic [21]. In this sense, other studies have also reported lower vitality scores associated with lower levels of physical activity, which negatively affects an individual’s well-being [22,23,24]. On the other hand, higher physical activity levels were associated with higher well-being and quality of life, and lower depressive symptoms, stress, and anxiety, regardless of age [25].

Concerning sports populations, adverse effects on athletes’ mental and emotional health during lockdown were reported [26]. More specifically, a recent study of recreational runners [11] also demonstrated that highly trained runners performed more and longer training sessions per week than lower-level runners during the lockdown. Moreover, in the first outdoor running session after confinement, highly trained runners performed a longer running session at a higher pace and covered a greater distance than lower-level runners, while enjoyment and motivation tended to be greater as runners’ level increased [11].

Well-being is considered an indicator of mental health [27]. Traditionally, well-being has been addressed from two well-differentiated perspectives [28]. On the one hand, the hedonistic perspective conceptualizes well-being as pleasure and affective experience, based on the presence of positive emotions and the absence of negative emotions [29]. On the other hand, eudaimonic well-being is oriented to meaning, excellence, and self-fulfillment, and represents the level at which an individual functions fully [30]. Thus, eudaimonic well-being is operationalized as a set of aspects that favor an individual’s well-being (pursuing intrinsic goals, behaving autonomously, and being mindful), satisfying basic psychological needs (i.e., autonomy, competence, and relatedness), and subjective vitality (SV) [31]. In this sense, SV refers to a positive feeling of aliveness and energy, where psychological and physiological factors converge [32], and has been considered a relevant outcome for evaluating psychological well-being [33]. In this regard, a recent review showed that a desirable health status is related to specific health behaviors (i.e., regular endurance and resistance training, sleep quality, and healthy nutrition) and to four psychological determinants (i.e., positive emotions, healthy mindsets, purposeful living, and social connectivity) that jointly interact to promote SV [34]. Thus, SV is positively related to general indicators of well-being, health, and moderate-intensity physical activity [35]. This is why individuals who perceive higher levels of energy are more likely to better manage stress, confront obstacles, pursue goals, or engage with the environment in other adaptive ways [36].

Recent scientific evidence suggests that participation in sports, at both recreational and elite levels, is related to better mental health. Specifically, sports participation promotes psychological well-being (e.g., higher self-esteem and life satisfaction), reduces psychological ill-being (e.g., reduced levels of depression, anxiety, and stress) and enhances social outcomes (e.g., pro-social behavior, self-control, interpersonal communication, etc.) [37]. Therefore, the practice of physical activity during the pandemic has proven to be an effective strategy for recovering people’s subsequent well-being [38]. However, some studies have suggested that the benefits of physical activity are influenced by the frequency and intensity of activity. The WHO recommends performing at least 150 to 300 min per week of moderate-to-vigorous physical activity to obtain health benefits, although lower levels can also positively affect health status [39]. The intensity and frequency of sports practice are associated with SV and psychological well-being, revealing that these individual traits are related to health and well-being in specific environments (e.g., physical exercise practice) [40]. Some studies have shown that running at high intensity and long-term training is associated with various positive mental health outcomes [41]. More specifically, previous studies have shown that recreational runners’ motivations to run are mainly related to eudaimonic well-being (e.g., maintain or improve health status, reach personal goals, and have fun) [42,43]. In this regard, motivation of recreational runners seems to be high in task-related goals (i.e., personal goal and mastery achievement) and low in ego-related goal orientation (i.e., competition and external achievement/social recognition) [42], while enjoyment and motivation for performance tend to be higher as the runner’s level increases [11]. Taken together, scientific evidence suggests that physical activity levels during the COVID-19 pandemic might play a key role in different-level athletes’ health status and sport-specific performance in the short- and long-term. In this sense, it was suggested that physical activity performed during lockdown (e.g., home-based training, quarantine training camps) attenuated the deleterious effects of the COVID-19 pandemic [26]. However, the volume (duration), frequency (days per week), and intensity of physical activity needed to achieve health benefits is still unknown, especially when mental health outcomes are considered. This is of great importance since analyzing training characteristics after an unprecedented situation such as a lockdown and relating them to SV outcomes might shed some light on the effects of regular physical activity on mental health and the optimal dose–response relationship.

Therefore, the aim of our study was to evaluate the sporting habits of recreational runners (frequency and duration of training sessions) and SV after the COVID-19 pandemic-induced confinement in Spain. To the best of our knowledge, no studies have addressed these issues in recreational runners after home confinement. Based on previous studies that have established a strong relationship between exercise and mental health [34,37,44,45,46], we hypothesized that runners who accumulate a greater weekly training volume (i.e., greater weekly training frequency and longer training session duration) would obtain higher scores in SV outcomes until an optimal dose–response relationship was reached. We consider our study will help coaches and athletes to better manipulate the training variables in order to optimize both health status and athletic performance among different-level runners.

## 2. Materials and Methods

### 2.1. Study Design and Data Collection

Our investigation was a cross-sectional study that included a self-reported questionnaire to assess participants’ sporting habits and SV after the COVID-19 pandemic-induced lockdown in Spain. The study was approved by the local university Ethics Committee (ETK-52/21-22) and personal informed consent was obtained when the questionnaire was completed and submitted by the participants. The participants were contacted through the database of RUNNEA’s web page (www.runnea.com, accessed on 15 September 2024), a company focused on training recreational runners and analyzing running and lifestyle shoes. In order to reach as many people as possible, the company’s main social networks (Facebook, Instagram, X, and LinkedIn) were used to promote participation in the study. Data were collected between 18 and 29 November 2020. After preliminary questions regarding sporting habits, the validated Spanish version [33] of the Subjective Vitality as a Dynamic Reflection of Well-Being (SVS) questionnaire [32] was sent via Google Forms. The reliability showed an α of Cronbach index of 0.86. This questionnaire is composed of six items containing a six-point Likert response scale (i.e., 1 = not at all, 4 = somewhat, and 7 = very true) and was designed to measure SV, reflecting the degree to which a person is fully functioning and psychologically well [33]. The total score ranges from 6 to 42 points, with a higher score indicating a better condition [47]. The theoretical model was tested using the weighted least squares method with LISREL 8.80. The root-mean-squared error of approximation (RMSEA), comparative fit index (CFI), non-normative fit index (NNFI), and standardized residual mean root (SRMR) were used to evaluate the goodness of fit. CFI and NNFI values greater than 0.90, and RMSEA and SRMR values lower than 0.08, reflect an acceptable fit. We also used a one-factor model. Given that the items belonged to the same factor, measurement errors were allowed to correlate. The four-factor solution showed good fit, X^2^ (714, n = 5542) = 1628, RMSEA = 0.076 (90% CI [0.072, 0.079], *p* = 0.36, CFI = 0.99, NNFI = 0.99.

### 2.2. Participants

All participants volunteered to take part in this study and were previously informed of the aim of the investigation, data recording techniques, and data analyses. Personal data privacy was guaranteed in accordance with the European General Data Protection Regulation (EU GDPR; EU 2016/679). The inclusion criteria were to be over 18 years old and to run at least 1–2 days per week. The exclusion criteria were incomplete or incongruent, as well as duplicate responses. After the 12-day period for data collection, data from 5549 recreational runners were registered. Data filtration was performed by two researchers, and once the inclusion and exclusion criteria were applied, 5542 recreational runners (4126 men and 1416 women) were included for further data analyses. Table 1 shows the sample sports habits according to sex and age group.

### 2.3. Procedures

Before the SVS questionnaire, the participants answered questions related to their sporting habits, which were included in the SVS questionnaire. Participants answered questions about (a) age group and sex, (b) training session duration (minutes), and (c) weekly training frequency (days). In this regard, following the classification framework proposed by McKay et al. [48], the great majority of our runners could be categorized as tier 1 (i.e., recreationally active) or tier 2 (i.e., trained/developmental).

### 2.4. Statistical Analysis

The data homogeneity of variance test was performed using Levene’s test, while Kolmogorov–Smirnov, Cramer–von Mises, and Anderson–Darling tests were used to analyze if the variables were normally distributed. A confirmatory factor analysis was performed with LISREL 8.80 [49] to test the adequacy of the theoretical structure of the questionnaire for the present sample. A non-parametric Mann–Whitney U test was performed to analyze possible significant differences between men and women in the final SVS questionnaire score.

A multiple regression model was set with SVS as the dependent variable and age group, training session duration, training frequency, interaction of age group × training frequency, age group × training duration, and training duration × training frequency as possible predictors. The final regression model was selected using a stepwise forward and backward method. For internal validation, k-fold cross-validation (10 folds and 5 repetitions) was performed. Internal validation was performed to reduce possible overfitting of the model [50]. Additionally, the R packages dplyr [51] and caret [52] were used to identify possible outliers and improve the fitting of the regression model. Outliers were identified and removed from the multiple regression model when the absolute value of the studentized residual (SRE) was ≥3. Model performance was assessed using the root-mean-square error (RMSE) and Pearson’s R^2^.

ANOVA tests were set to establish potential interaction training frequency and mean duration of training sessions on the SVS. When the ANOVA test showed significant differences between factors, partial eta squared (η^2^) was used as a measure of effect size (ES), using the reference values of small (η^2^ = 0.01), medium (η^2^ = 0.06), and large (η^2^ = 0.14). A subsequent post hoc Tukey’s test was performed to compare the potential differences between factors. For significant differences, Cohen’s *d* was used as a measure of ES, using the reference values of small (*d* = 0.2), medium (*d* = 0.5), and large (*d* = 0.8) for interpreting them, as suggested by Cohen [53]. R software 4.2.2 (R Core Team, Vienna, Austria, 2022) and RStudio (version 2022.12.0.353; Rstudio Team, 2022) were used for statistical analysis. The significance level was established at a value of *p* ≤ 0.05.

## 3. Results

### 3.1. Sex and Age Groups: Questionnaire’s Final Scores

There were no significant differences between men and women regarding the questionnaire’s final scores (33.0 ± 5.67 vs. 34.0 ± 5.96 for men and women, respectively) (*p* > 0.05). Similarly, scores on the SVS questionnaire did not differ between the age groups (*p* > 0.05).

### 3.2. Regression Model

Once outliers were identified and removed, a final sample of 5478 (4080 men and 1398 women) was included for the final regression model and subsequent analysis. After stepwise regression, the final model considered training frequency, training session duration, and interaction between frequency and training duration as explanatory variables. There were no significant interactions between the other independent variables. The final regression model showed a significant (*p* < 0.001) but very small explanatory capacity (R^2^ = 0.08, 95%CI-0.06–0.09; RSE = 5.51).

Even though potential outliers were eliminated, analysis of the final regression model’s residuals revealed that they did not follow a normal distribution, but did fit the heteroscedasticity assumption. Thus, the final regression model was constructed using a generalized linear method (Table 2).

### 3.3. Effects of Sporting Habits on SVS

Three-way ANOVA was performed (frequency × duration × frequency: duration) (*p* = 0.005).

**Figure 1:**
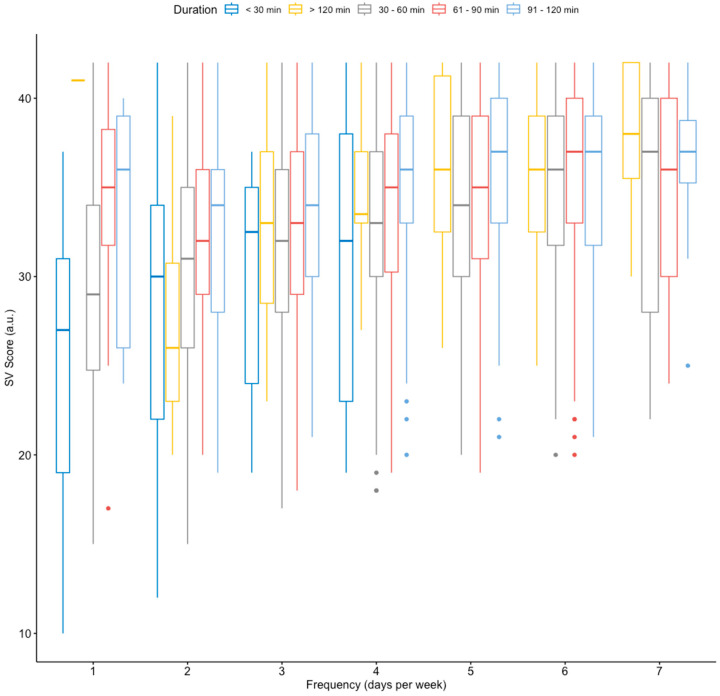
The box plot diagram illustrates the interaction between training frequency and training session duration on the SVS. Colored points represent potential outliers.

This ANOVA test reported significant differences in SVS based on training frequency (F (1, 6090) = 199.715, *p* < 0.001; ηp^2^ = 0.018), training session duration (F (4, 7272) = 59.62, *p* < 0.001; ηp^2^ = 0.035), and the interaction of these variables (F (4, 447) = 3.663, *p* < 0.001; ηp^2^ = 0.003). Figure 1 shows the interaction between training frequency and training session duration on the SVS. Similarly, Table 3 shows only the pairwise comparison (frequency × duration) for the significant differences in the SVS.

In Table 3, it could be observed that the largest ES (*d* = 0.8) occurred with increasing training frequency. Whereas longer training duration produced significant differences on SVS, but of a low or moderate nature (Table 3).

## 4. Discussion

To the best of our knowledge, this is the first study to evaluate recreational runners’ sporting habits and SV after the COVID-19 pandemic-induced lockdown. The main findings of our investigation were: (1) no differences were found for SV scores between men and women, nor between age groups; (2) SV improved with weekly training frequency up to 5 days/week, where this enhancement plateaued; and (3) average training session duration was positively related to SV, stabilizing at 91–120 min/session. Finally, our hypothesis was confirmed, showing a dose–response relationship between the frequency and duration of training sessions and SV scores in recreational runners.

### 4.1. Final Scores of the Questionnaire Regarding Sex and Age Groups

Concerning the questionnaire’s final scores, we found no significant differences between men and women, nor between age groups regarding SV. These results are not in accordance with previous studies that reported a higher risk for mental health outcomes in women [26], such as higher levels of perceived stress [5], while older and experienced athletes seem to have lower motivation scores [54], lower stress levels, and higher functional biopsychosocial states [5]. Our results could be partially explained by the heterogeneity of our sample regarding age groups, as well as by the lower number of female participants (25.5%) compared to previous studies (49–53%) [5,54]. Regarding this, in the last decades, the number of female runners substantially increased from under 20% in 1986 up to ~50% in 2018. However, participation rates differ between running distances, with a trend to decrease the number of female participants as the distance increases. On the other hand, women’s participation in running events is quite different worldwide. For instance, in countries such as the United States or Canada, the percentage of women is clearly above 50%, while in other European countries (e.g., Switzerland, Italy), female participation is under 20% [1].

### 4.2. Explanatory Variables

The final regression model showed sex, training frequency, training session duration, and interaction between sex and duration, and between frequency and duration as explanatory variables. Although the model’s explanatory capacity was significant, it can be considered very small (R^2^ = 0.08, 95%CI-0.06–0.09; RSE = 5.51). However, it is interesting to highlight that the explanatory variables had different weights in the equation. For instance, training session duration showed a greater weight (i.e., from 5.84 to 8.03) than training frequency (i.e., 2.03), which suggests that the training session duration might be related to the SVS scores in a greater manner. However, although our data showed that session duration has more impact on SVS than training frequency, other previous studies have considered training intensity in fitness maintenance for the general population a key role even though frequency and/or volume (i.e., duration) of exercise is reduced by 33–66% [55]. In this regard, we have not collected data related to PA intensity; however, it could be assumed that the majority of recreational runners performed vigorous-intensity PA (i.e., ≥6 metabolic equivalent task, METS) since, for instance, running at 4 mph or 6.5 km/h (i.e., 13 min/mile and 9 min/km, respectively) demands an exercise intensity of ~6 METS [56].

### 4.3. Subjective Vitality Effects on Recreational Runners’ Sporting Habits

The unprecedented situation derived from the COVID-19 pandemic forced athletes of different levels to face this challenging situation by modifying their training programs. Previous research reported important reductions in training loads, or even the cessation of training routines, thus leading to detraining that negatively affected athletes’ health status and performance level [11]. Beyond the situation of the COVID-19 pandemic, and despite scientific evidence consistently demonstrating the positive relationship between PA and well-being [37,57,58], there is a lack of knowledge about how much PA is needed to improve well-being and, specifically, SV (i.e., optimal dose–response relationship), especially in recreational runners of different levels. In this regard, the American College of Sports Medicine (ACSM) recommends following the FITT-VP principle of exercise prescription, which includes frequency (i.e., how often exercise is performed per week), intensity (i.e., how hard exercise is), time (i.e., exercise duration), type (i.e., exercise mode), volume (i.e., total amount of exercise), and progression (i.e., advancement of the training program) [59]. Accordingly, the WHO recommends, in its updated guidelines on PA and sedentary behavior for healthy adults, a minimum weekly accumulation of 150–300 min of moderate-intensity aerobic PA or 75–150 min of vigorous-intensity aerobic PA for maintaining or enhancing healthy fitness [60]. Nevertheless, to the best of our knowledge, only a few studies [5,15] have focused on the fluctuation of well-being in athletes before and after lockdowns in two of the countries (Spain and Italy) most affected by the COVID-19 pandemic. Martínez-González and colleagues [15] showed a negative impact of the COVID-19 lockdown on Spanish university athletes’ SV and autonomous goal motives (i.e., task-related goal orientation). Similarly, di Fronso et al. [5] reported higher levels of perceived stress and dysfunctional biopsychosocial states during the pandemic. In this regard, it was suggested that a reduction in performed PA and exercise during lockdown could be related to lower vitality scores, thus leading to a loss of well-being in the physically active population [22,23,24]. However, scientific evidence does not provide a specific recommendation about the minimum exercise frequency or duration (i.e., training session time) for maximizing mental health benefits, especially when SV in different-level recreational runners is considered. Our study could shed some light on this issue since our results showed that there was a positive relationship between SVS scores and training frequency up to 5 days/week, whereas this relationship was interrupted with higher training frequencies (Figure 1). Moreover, runners who performed the fewest training sessions during the week also reported the worst SVS scores (Table 3). As can be seen in Table 3, a higher training frequency reported a higher SVS score when ES was taken into account. Therefore, it seems that frequency is one of the main components to be taken into account when improving the SVS score. At the same time, an increase in training duration did not produce ES as high as those achieved with higher training frequency. For this reason, there seems to be a threshold at which a higher weekly training frequency is not related to greater SVS scores in recreational athletes. However, future longitudinal studies are needed to confirm these results.

Similarly, despite scientific evidence strongly supporting the implementation of regular PA and exercise as an effective strategy for improving various psychological disorders [61,62] and well-being [58,63], there are no specific guidelines, beyond the general recommendations of the WHO [60], regarding the optimal dose–response of exercise duration for optimizing the prognosis of these disorders. In this context, our study shows that there is a positive relationship between running training session duration and SVS scores. However, this tendency stabilizes when the session duration exceeds 61–90 min. Thus, our results suggest that training session times above 91–120 min are not related to higher SVS scores (Figure 1). Nevertheless, these results should be interpreted with caution since only a few recreational runners (5.1%) reported performing training sessions longer than 90 min, and the ES was small. Therefore, a duration of 61–90 min per session is likely to produce similar results to those of a longer duration. Related to this, although a minimum period of time seems to be necessary to obtain some psychological benefits, the minimum duration per session remains unclear. Hoffman and Hoffman [63] demonstrated that a single 20-min session improved the final score in the Profile of Mood States (POMS) in physically active individuals (3–6 sessions per week, 30–60 min of aerobic exercise) and ultramarathon runners. However, these authors reported no improvements in the perception of vitality or fatigue in non-exercisers. These data highlight the importance of performing PA on a regular basis for obtaining health benefits, since an isolated bout of exercise seems insufficient in physically inactive and/or sedentary individuals. In this context, our analysis did not allow us to determine a minimum time (i.e., duration) of effective practice or a minimum number of weekly training sessions to improve SV and, therefore, mental health. Nevertheless, our initial hypothesis has been confirmed, as a dose–response relationship between frequency and duration of training and an increased perception of SV has been demonstrated.

## 5. Future Research Lines and Limitations

One of the limitations of our study is the administration of a self-reported questionnaire, which can lead to recall bias. Nevertheless, we consider our study reports low recall bias, since our participants provided responses of a frequent event or routine (i.e., daily training habits), thus the recall period was short. Another limitation is the fact that we did not quantify different-intensity PA performed before and during the pandemic using a validated questionnaire (e.g., IPAQ). However, we have obtained data related to the majority of FITT-VP parameters suggested by the ACSM (i.e., training frequency, training session time or duration, exercise type or modality, and training volume). Further, one could assume that the majority of recreational runners performed vigorous-intensity PA (i.e., ≥6 METS). In this regard, the main aim of our study was to assess the effects of different weekly training volume (i.e., weekly training frequency × training session duration), regardless of exercise intensity, on SV in recreational runners of different levels after lockdown. Our results suggest that there is an optimal dose–response relationship in terms of weekly training frequency and training session duration for maximizing well-being in different-level recreational runners (mainly tiers 1 and 2). These results may be not be extended to runners of a higher level of performance (e.g., tiers 3 to 5), since the necessary training stimuli for optimizing both athletic performance and well-being may differ, thus resulting in a different dose–response relationship.

Since one cannot discard more similar situations in the future, like social isolation, it would be interesting to perform longitudinal studies to determine what is the optimal PA intensity and the optimal combination of PA intensity, training frequency, and training session duration for maximizing mental health benefits in runners of different levels. This would help runners and coaches to properly manipulate endurance training variables for simultaneously maintaining or enhancing health status and endurance performance. Further, this information could be of great importance for governments to optimize public health policies according to the specific scenario of a region in order to maintain, or even improve, the population’s health status. On the other hand, another limitation of the present study was the heterogeneity of our sample and the small number of runners who trained more than 5 days per week and more than 90 min per session. Thus, our results should be interpreted with caution since regarding sex, there was a considerable difference in the proportion of participants (men 74.5% and women 25.5%) who took part in this investigation, beyond the fact that our investigation was a cross-sectional study. In this sense, future research strategies should focus on promoting a higher women participation rate. Finally, we could not control any potential confounding variables (e.g., injuries, cardiovascular events) that might have affected our results.

## 6. Conclusions

This is the first study to evaluate the influence of training session duration and training frequency on SV in different-level recreational runners. Based on our results, SV was greater with a weekly training frequency of up to 5 days/week, where this enhancement plateaued, while average training session duration was positively related to SV, stabilizing at 91–120 min/session. We conclude that there is a dose–response relationship between both weekly training frequency and training session duration and mental health benefits in recreational runners. Future longitudinal studies are needed to determine the optimal dose–response relationship for simultaneously maximizing mental health benefits and endurance performance in recreational runners, especially regarding weekly training frequency, training session duration, and exercise intensity.

## Figures and Tables

**Table 1:** Sample sporting habits regarding sex and age group.

				Sporting Habits										
Age Group (Years)	Sex	n, % Total		<30 min (n, %)		30–60 min (n, %)		61–90 min (n, %)		91–120 min (n, %)		>120 min (n, %)		Weekly Training Frequency (Days) (Mean ± SD)
<19	Female	9	0.16%	0	0.0%	2	0.0%	6	0.1%	1	0.0%	0	0.0%	4.33 ± 1.50
	Male	31	0.56%	1	0.0%	14	0.3%	10	0.2%	1	0.0%	5	0.1%	4.23 ± 1.69
19–25	Female	142	2.56%	5	0.1%	84	1.5%	42	0.8%	10	0.2%	1	0.0%	3.55 ± 1.32
	Male	264	4.76%	11	0.2%	147	2.7%	86	1.6%	18	0.3%	2	0.0%	3.77 ± 1.32
26–30	Female	206	3.72%	5	0.1%	137	2.5%	57	1.0%	7	0.1%	0	0.0%	3.28 ± 1.24
	Male	313	5.65%	7	0.1%	172	3.1%	119	2.1%	14	0.3%	1	0.0%	3.55 ± 1.24
31–35	Female	214	3.86%	10	0.2%	127	2.3%	65	1.2%	12	0.2%	0	0.0%	3.36 ± 1.31
	Male	502	9.06%	6	0.1%	281	5.1%	194	3.5%	19	0.3%	2	0.0%	3.49 ± 1.16
36–40	Female	225	4.06%	4	0.1%	134	2.4%	75	1.4%	9	0.2%	3	0.1%	3.32 ± 1.26
	Male	686	12.38%	8	0.1%	370	6.7%	278	5.0%	26	0.5%	4	0.1%	3.45 ± 1.12
41–45	Female	289	5.21%	9	0.2%	141	2.5%	124	2.2%	13	0.2%	2	0.0%	3.37 ± 1.18
	Male	937	16.91%	8	0.1%	438	7.9%	435	7.8%	52	0.9%	4	0.1%	3.45 ± 1.14
46–50	Female	186	3.36%	3	0.1%	79	1.4%	92	1.7%	8	0.1%	4	0.1%	3.30 ± 1.14
	Male	757	13.66%	8	0.1%	343	6.2%	352	6.4%	52	0.9%	2	0.0%	3.59 ± 1.16
51–55	Female	96	1.73%	1	0.0%	40	0.7%	49	0.9%	5	0.1%	1	0.0%	3.38 ± 1.01
	Male	395	7.13%	9	0.2%	158	2.9%	197	3.6%	24	0.4%	7	0.1%	3.59 ± 1.17
56–60	Female	36	0.65%	1	0.0%	16	0.3%	15	0.3%	4	0.1%	0	0.0%	3.31 ± 1.37
	Male	162	2.92%	3	0.1%	60	1.1%	84	1.5%	11	0.2%	4	0.1%	3.72 ± 1.35
61–65	Female	9	0.16%	1	0.0%	6	0.1%	2	0.0%	0	0.0%	0	0.0%	2.56 ± 0.73
	Male	50	0.90%	0	0.0%	13	0.2%	30	0.5%	7	0.1%	0	0.0%	3.92 ± 1.14
66–70	Female	4	0.07%	0	0.0%	1	0.0%	2	0.0%	0	0.0%	1	0.0%	5.00 ± 1.41
	Male	25	0.45%	0	0.0%	6	0.1%	14	0.3%	2	0.0%	3	0.1%	4.08 ± 1.35
>70	Female	0	0.0%	0	0.0%	0	0.0%	0	0.0%	0	0.0%	0	0.0%	-
	Male	4	0.07%	0	0.0%	2	0.0%	2	0.0%	0	0.0%	0	0.0%	3.75 ± 0.96

Data are presented in counts (n) and percentages of total (%), as well as mean and standard deviation (mean ± SD), when applicable.

**Table 2:** Regression model results.

Predictor	b	Std. Error	t-Value
Intercept	23.74 **	1.35	17.54
Frequency (days per week)	2.03 **	0.58	3.49
Duration > 120 min	5.84 *	2.72	2.15
Duration 30–60 min	4.63 **	1.39	3.33
Duration 61–90 min	7.36 **	1.41	5.22
Duration 91–120 min	8.03 **	1.70	4.72
Frequency: Duration > 120 min	−0.92	0.76	−1.21
Frequency: Duration 30–60 min	−0.88	0.59	−1.49
Frequency: Duration 61–90 min	−1.33 *	0.59	−2.25
Frequency: Duration 91–120 min	−1.28 *	0.63	−2.03

Note. A significant *b*-weight indicates that the semi-partial correlation is also significant. b represents unstandardized regression weights = represents the interaction between the factors. * indicates *p* < 0.05. ** indicates *p* < 0.01.

**Table 3:** Comparison of pairwise analysis between training session duration and training frequency.

Training Session Duration	Training Frequency (Days)	SVS ± SD		Training Frequency (Days)	SVS ± SD	*p.* adj.	ES (*d*)
30–60 min	1	29.0 ± 6.47	VS.	3	32.0 ± 5.67	<0.001	0.52
				4	33.1 ± 5.30	<0.001	0.75
				5	34.1 ± 5.36	<0.001	0.89
				6	34.7 ± 5.43	<0.001	0.94
				7	34.8 ± 6.72	<0.001	0.89
30–60 min	2	29.0 ± 6.48		3	30.5 ± 6.02	<0.001	0.24
				4	33.1 ± 5.30	<0.001	0.7
				5	34.1 ± 5.36	<0.001	0.82
				6	34.7 ± 5.43	<0.001	0.89
				7	34.8 ± 6,72	<0.01	0.89
30–60 min	3	32.0 ± 5.67		4	33.1 ± 5.30	<0.001	0.2
				5	34.1 ± 5.36	<0.001	0.37
				6	34.7 ± 5.43	<0.001	0.48
61–90 min	2	32.3 ± 6.02		4	34.0 ± 5.15	<0.001	0.32
				5	34.6 ± 5.21	<0.001	0.42
				6	35.7 ± 5.55	<0.001	0.59
61–90 min	3	33.1 ± 5.33		5	34.6 ± 5.21	<0.001	0.28
				6	35.7 ± 5.55	<0.001	0.48
61–90 min	4	34.0 ± 5.15		6	35.7 ± 5.55	<0.001	0.33
91–120 min	2	32.0 ± 6.34		5	36.2 ± 4.98	<0.01	0.79

Note. For a better understanding, the present table only shows comparisons of significant differences. SVS: Subjective Vitality as a Dynamic Reflection of Well-Being; ES: effect size; SD: standard deviation.

## Data Availability

The data presented in this study are available on request from the corresponding author due to ethical reasons.
